# Supplementary material for: Cancer stem cell regulated phenotypic plasticity protects metastasized cancer cells from ferroptosis
Source: Nat Commun. 2022 Mar 16;13:1371. doi: 10.1038/s41467-022-29018-9 (PMC8927306; doi:10.1038/s41467-022-29018-9)
Supplement: Supplementary file 1 — Supplementary tables and Figures [file 41467_2022_29018_MOESM1_ESM.pdf]

Supplementary Fig. 1

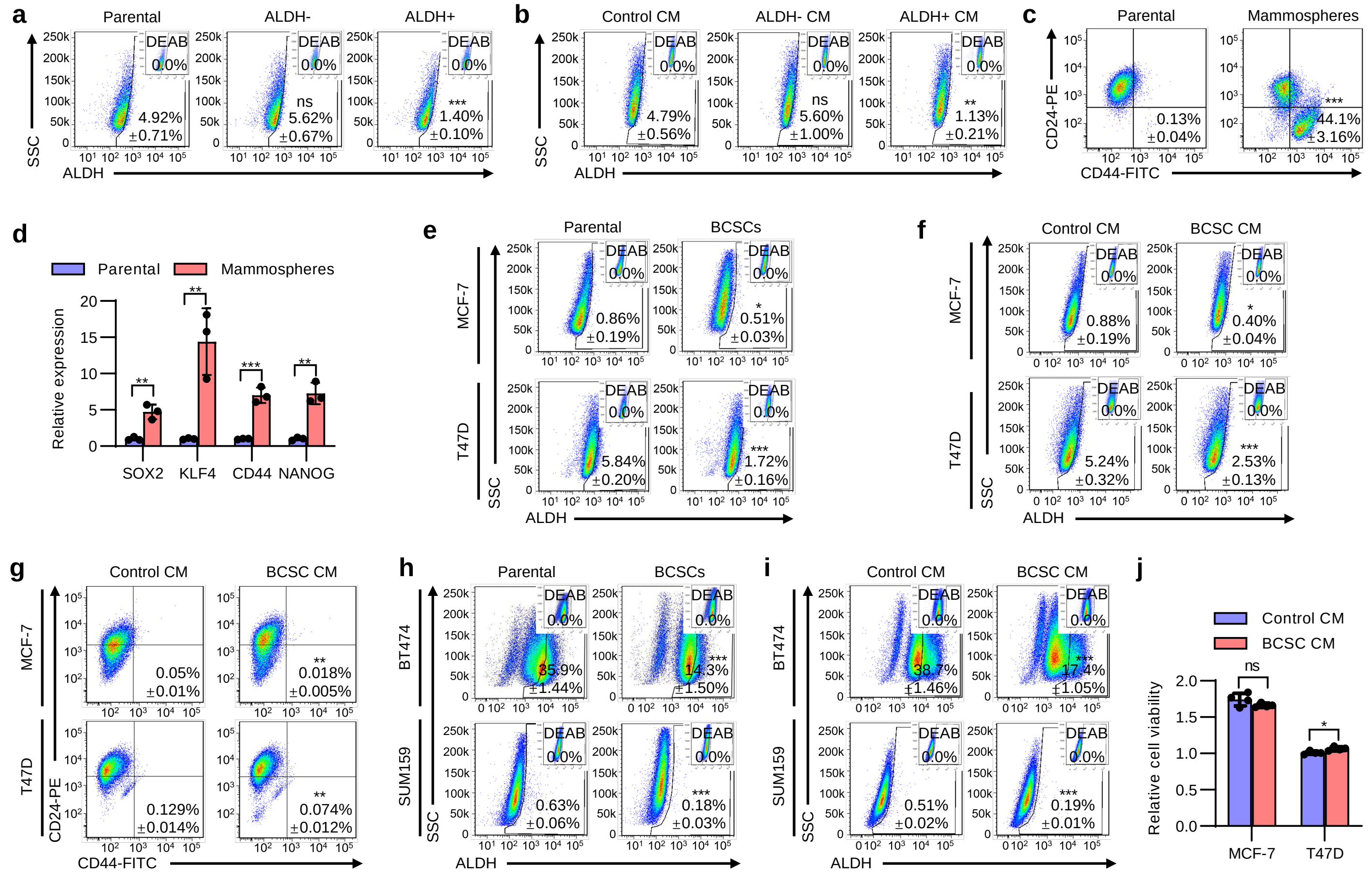

Supplementary Fig. 2

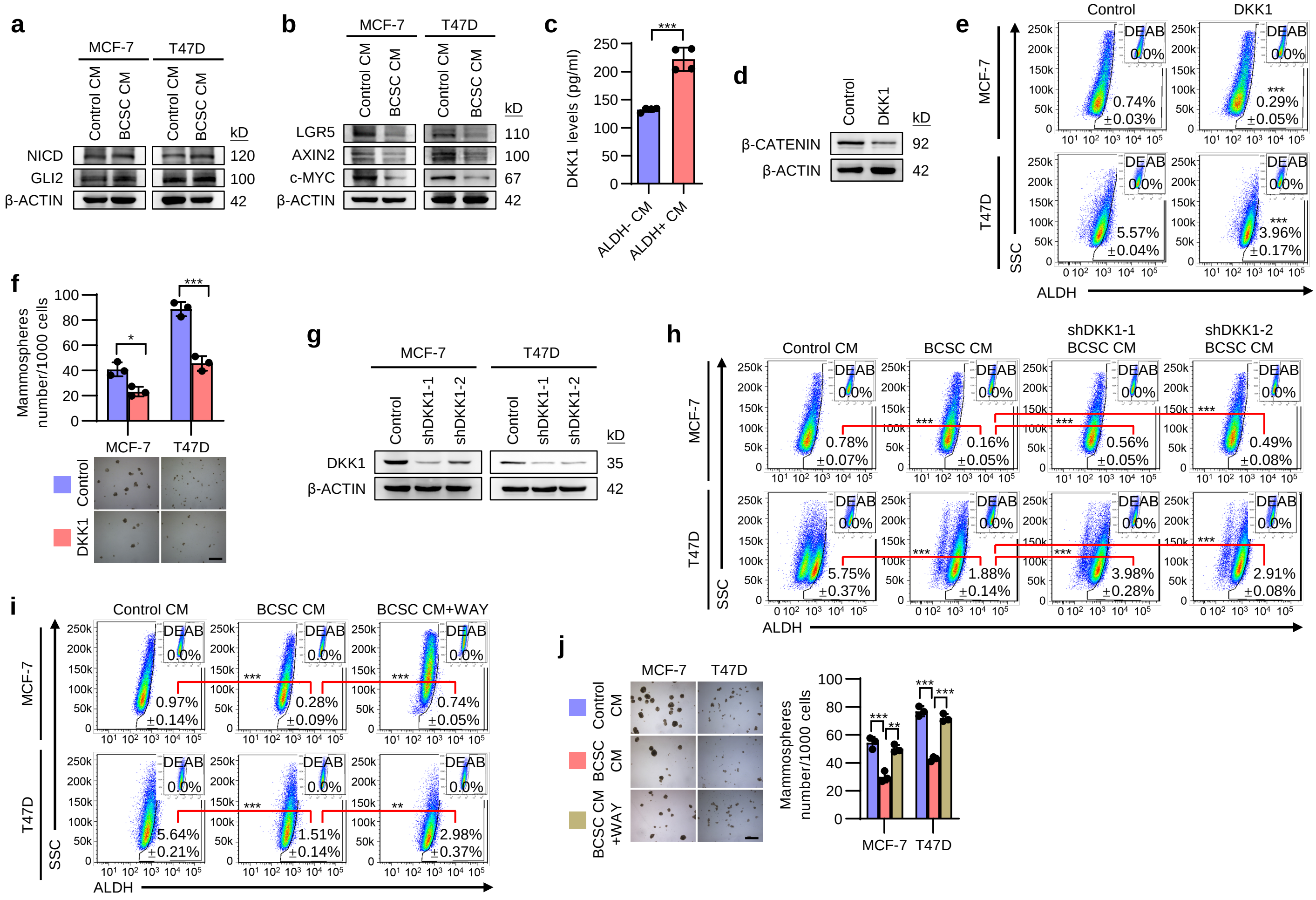

Supplementary Fig. 3

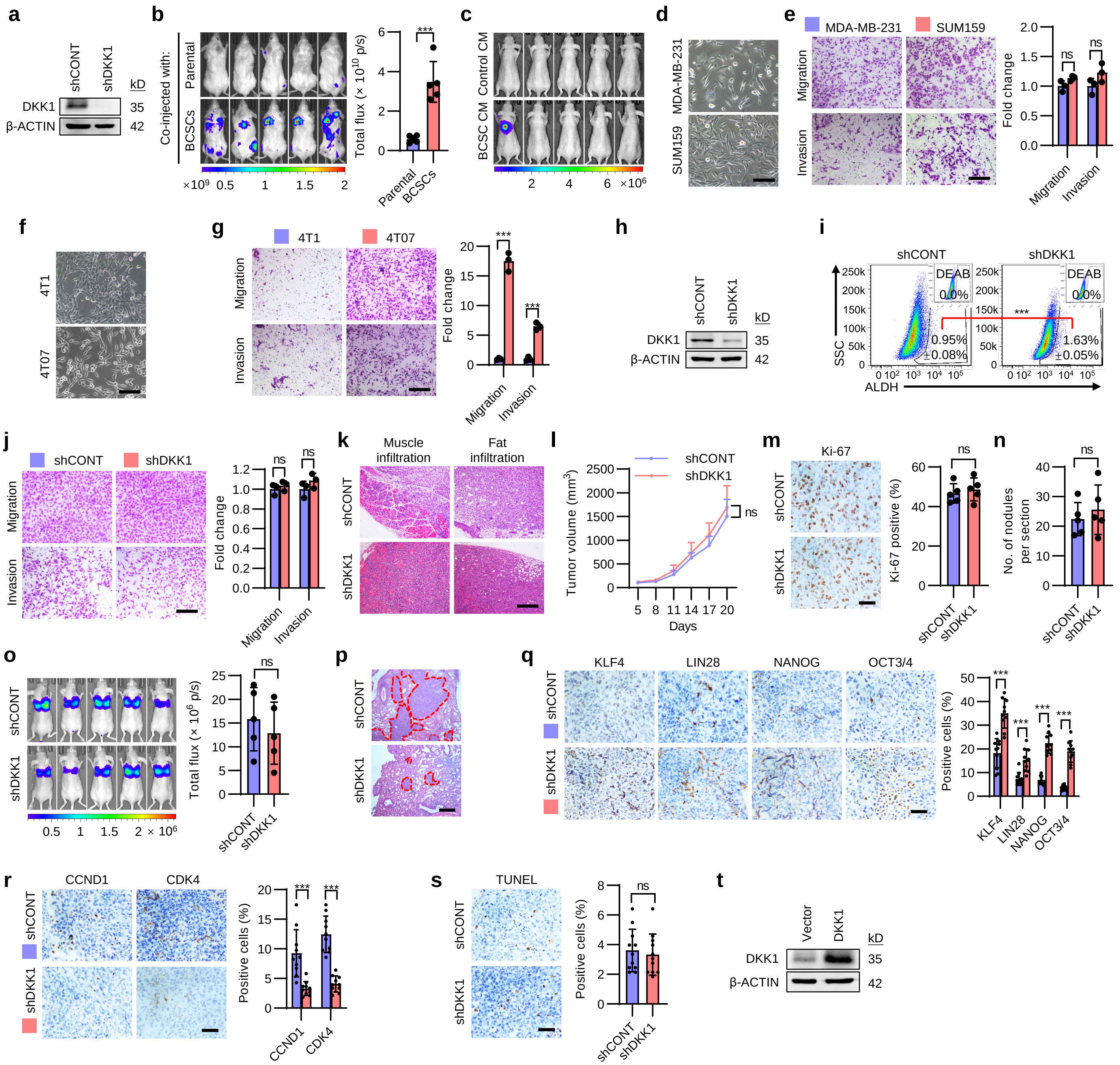

Supplementary Fig. 4

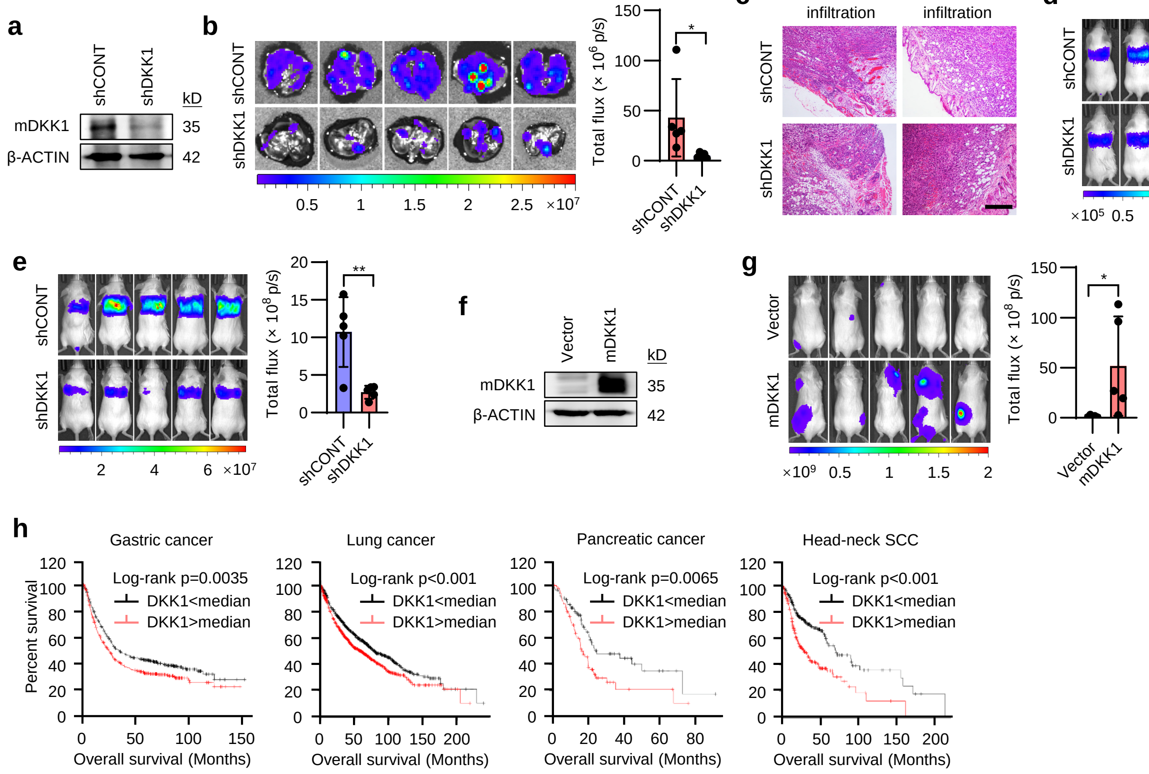

Supplementary Fig. 5

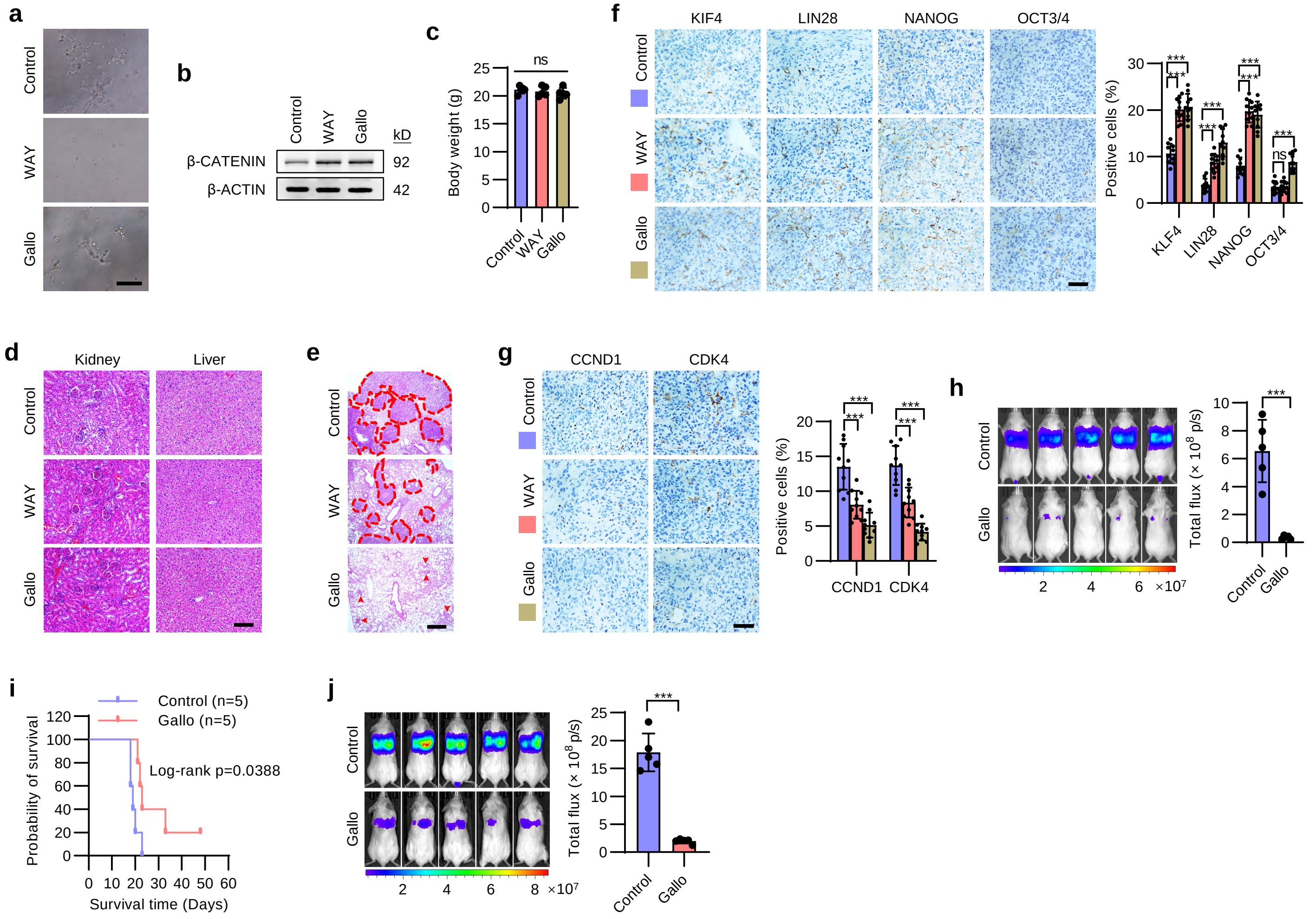

Supplementary Fig. 6

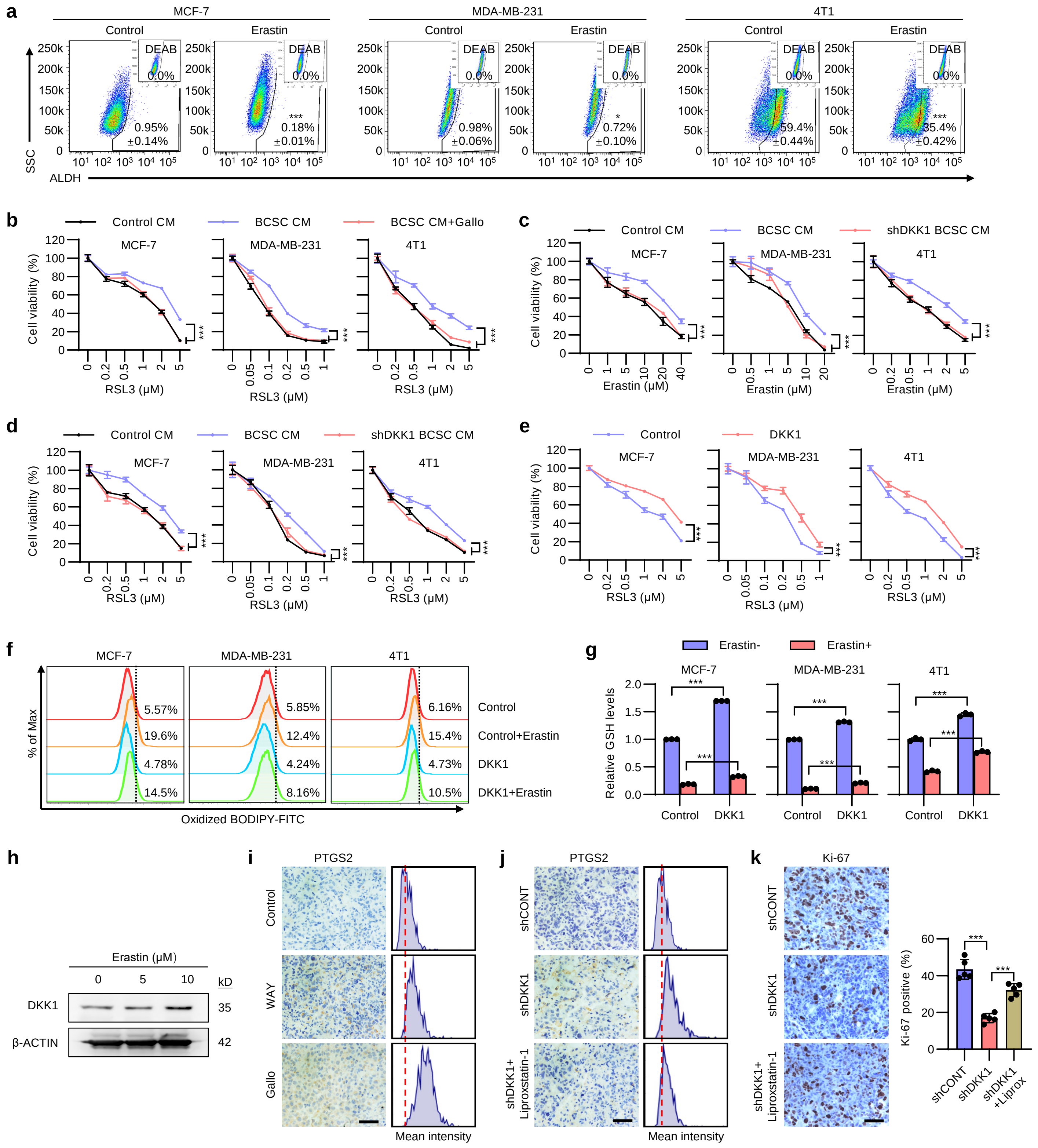

Supplementary Fig. 7

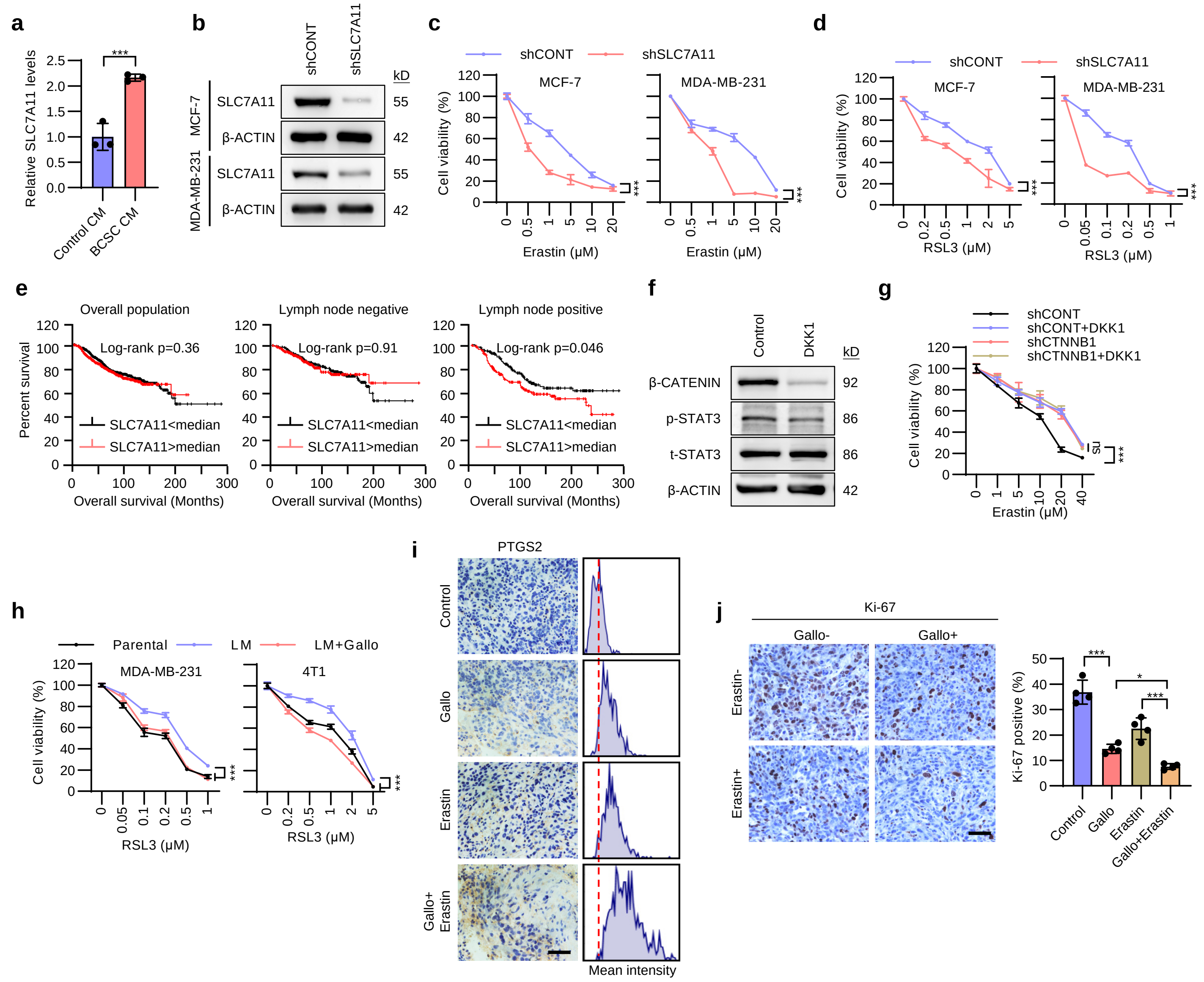

Supplementary Fig. 8

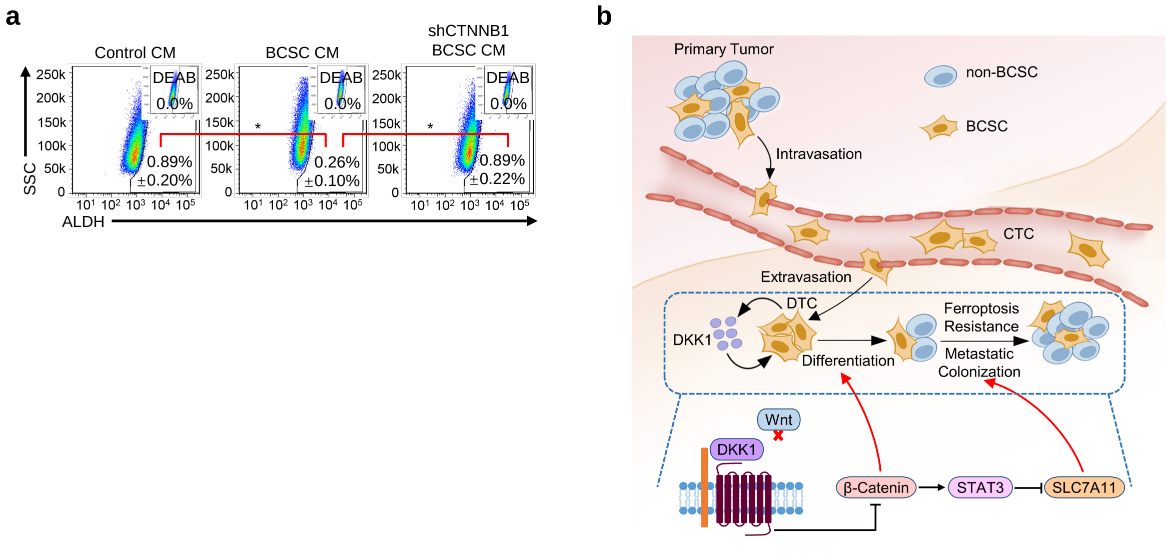

Supplementary Table 1 Hematological and blood biochemical indices in WAY262611 or Gallocyanine treated BALB/c mice. The BALB/c mice were eated with saline, WAY262611 or Gallocyanine for 3 weeks. Mice were sacrificed and hematological and blood biochemical parameters were analyzed. The values are shown as mean ± S.D. Statistical significance was determined by one-way ANOVA followed by Tukey's multiple comparison test (versus saline treated group).

Hematological parameter	Control n=4	WAY26261 n=5	Gallocyanine n=5
White blood cell WBC (10^9/L)	7.43 ± 2.00	7.23 ± 0.67 p=0.971	9.75 ± 1.63 p=0.071
Neutrophilic granulocyte NEUT (10^9/L)	1.20 ± 0.38	1.20 ± 0.21 p>0.999	1.31 ± 0.41 p=0.835
Lymphocytes LYMPH (10^9/L)	6.21 ± 1.62	5.99 ± 0.67 p=0.950	8.36 ± 1.36 p=0.045 *
Monocytes MONO (10^9/L)	0.02 ± 0.008	0.028 ± 0.008 p=0.587	0.066 ± 0.019 p=0.001 ***
Eosnophils EO (10^9/L)	0.003 ± 0.005	0.006 ± 0.009 p=0.646	0.002 ± 0.004 p=0.990
Basophils BASO (10^9/L)	0.01 ± 0.00	0.01 ± 0.007 p>0.999	0.014 ± 0.005 p=0.453
Red blood cell RBC (10^{12}/L)	10.56 ± 0.25	10.06 ± 0.26 p=0.034 *	10.09 ± 0.29 p=0.044 *
Hemogloblin HGB (g/L)	161.5 ± 3.51	154.6 ± 3.91 p=0.061	153.4 ± 5.08 p=0.030 *
Hematocrit HCT (%)	56.20 ± 1.61	52.10 ± 1.44 p=0.007 **	52.08 ± 1.92 p=0.007 **
Mean corpuscular volume MCV (fL)	53.28 ± 0.80	51.78 ± 0.49 p=0.022 *	51.60 ± 0.90 p=0.012 *
Mean corpuscular hemogloblin MCH (pg/cell)	15.30 ± 0.16	15.36 ± 0.29 p=0.882	15.22 ± 0.16 p=0.804
Mean corpuscular hemogloblin concentration MCHC (g/L)	287.5 ± 2.08	296.8 ± 5.36 p=0.009 **	294.4 ± 3.36 p=0.044 *
Platelets PLT (10^9/L)	684.5 ± 30.2	727 ± 66.5 p=0.468	725 ± 66.2 p=0.498
Plateletcrit PCT (%)	0.518 ± 0.015	0.534 ± 0.045 p=0.754	0.576 ± 0.045 p=0.083
Platelet distribution width PDW (fL)	8.20 ± 0.61	8.48 ± 1.15 p=0.832	9.96 ± 0.55 p=0.017 *
Mean platelet volume MPV (fL)	7.55 ± 0.10	7.38 ± 0.41 p=0.663	8.00 ± 0.35 p=0.115
Platelet larger cell ratio P-LCR (%)	11.75 ± 1.26	10.00 ± 3.32 p=0.545	15.00 ± 2.92 p=0.176
Blood biochemical parameters	Control n=3	WAY26261 n=3	Gallocyanine n=3
Alanine aminotransferase ALT (U/L)	41.67 ± 4.04	44.00 ± 5.57 p=0.905	161.00 ± 11.27 p<0.0001 ***
Aspartate aminotransferase AST (U/L)	107.33 ± 22.50	171.00 ± 67.64 p=0.229	264.33 ± 34.56 p=0.010 *
Alkaline phosphatase ALP (U/L)	185.67 ± 3.79	184.33 ± 10.60 p=0.984	279.33 ± 15.63 p<0.0001 ***
Lactate dehydrogenase LDH (U/L)	2047.3 ± 797.7	2541.7 ± 1093.6 p=0.677	3197.6 ± 140.9 p=0.204
Urea UREA mmol/L	8.00 ± 1.40	8.13 ± 0.32 p=0.986	8.40 ± 1.45 p=0.885
Creatinine CRE (μmol/L)	21.97 ± 4.61	18.17 ± 1.20 p=0.408	19.13 ± 4.38 p=0.580
Uric acid UA (μmol/L)	77.67 ± 15.95	115.33 ± 30.99 p=0.151	88.33 ± 18.58 p=0.798
Creatinine kinase CK (U/L)	881.7 ± 293.7	1794.3 ± 1303.2 p=0.343	1047.7 ± 402.7 p=0.955

Supplementary Table 2 A summary of information about the clinical characteristics, gender, age, tumor stage and molecular subtype of patients.

Patient	Gender	Age	Cancer type	Tumor stage	ER	PR	HER2	Tumor site
1	Female	52	Breast cancer	II	90%	70%	Negative	Left
2	Female	58	Breast cancer	III	Negative	Negative	3+	Left
3	Female	52	Breast cancer	II	90%	80%	1+	Right
4	Female	48	Breast cancer	III	Negative	Negative	3+	Left
5	Female	47	Breast cancer	III	40%	40%	3+	Right
6	Female	54	Breast cancer	II	90%	80%	2+	Right
7	Female	43	Breast cancer	II	85%	80%	3+	Right
8	Female	71	Breast cancer	II	90%	30%	Negative	Left
9	Female	62	Breast cancer	II	90%	20%	2+	Left
10	Female	57	Breast cancer	III	Negative	Negative	3+	Right
11	Female	55	Breast cancer	III	90%	5%	1+	Left
12	Female	55	Breast cancer	II	90%	10%	1+	Left
13	Female	70	Breast cancer	II	90%	90%	1+	Left
14	Female	39	Breast cancer	II	90%	90%	Negative	Right
15	Female	44	Breast cancer	II	Negative	Negative	3+	Left

Supplementary Table 3 Sequences of shRNAs, and primers for cloning, ChIP assays, and qRT-PCR analysis.

shRNAs	Sense Strand (5'-3')	
DKK1 shRNA1	CCGGCGGTTCTCAATTCCAACGCTACTCGAGTAGCGTTGGAATTGAGAACCGTTTTTG	
DKK1 shRNA2	CCGGCCTGTCCTGAAAGAAGGTCAACTCGAGTTGACCTTCTTTCAGGACAGGTTTTTG	
β-CATENIN shRNA1	CCGGAGGTGCTATCTGTCTGCTCTACTCGAGTAGAGCAGACAGATAGCACCTTTTTT	
β-CATENIN shRNA2	CCGGGCTTGGAATGAGACTGCTGATCTCGAGATCAGCAGTCTCATTCCAAGCTTTTT	
SLC7A11 shRNA	CCGGCCTGCGTATTATCTCTTTATTCTCGAGAATAAAGAGATAATACGCAGGTTTTTG	
mDKK1 shRNA	CCGGGATCACCATCAAGCCAGCAATGGATCCATTGCTGGCTTGATGGTGATCTTTTTG	
Primers for plasmid construction	Sense Strand (5'-3')	Antisense Strand (5'-3')
Firefly luciferase cloning primer	CCGAATTCATGGCCGATGCTAAGAACATTA	AAGGATCCTTACACGGCGATCTTGCCGCCT
DKK1 (Homo) cloning primer	ATGAATTCATGATGGCTCTGGGCGCAGCG	GCGGATCCTTAGTGTCTCTGACAAGTGTG
DKK1 (Mus) cloning primer	GGAATTCATGATGGTTGTGTGTGCAGCG	AAGGATCCTTAGTGTCTCTGGCAGGTGTG
DKK1 promoter cloning primer	GGCCGCTAGCAGATTCTGTCCAGACTCAGTGA	ATATCTCGAGCGCCGCCACCGCCACCGCGGCT
DKK1 promoter mut1 primer	GCTTTGGTCCCGGCCCTCACGCGTCTGCCTAATCAAGTTC	AGGCAGACGCGTGAGGGCCGGGACCAAAGCGGACAGGGTC
DKK1 promoter mut2 primer	CCAGCCCCTCCCAGCATCCCATCCCGGCTTTGTTGTCTCC	AAGCCGGGATGGGATGCTGGGAGGGGCTGGGAGGGGGTGTC
Primers for ChIP assay	Sense Strand (5'-3')	Antisense Strand (5'-3')
DKK1 ChIP	CCGGCCACTTTGATCTCAC	GACCATCCGATAATCAAACCA
GAPDH ChIP	TACTAGCGGTTTTACGGGCG	TCGAACAGGAGGAGCAGAGAGCGA
Primers for qRT-PCR	Sense Strand (5'-3')	Antisense Strand (5'-3')
GAPDH	TGCACCACCAACTGCTTAGC	GGCATGGACTGTGGTCATGAG
SOX2	GGGGGAATGGACCTTGTATAG	GCAAAGCTCCTACCGTACCA
KLF4	ACATGGCTGTCAGCGACGCG	GCCAGCGGTTATTCGGGGCAC
CD44	CAGGGACAGCTGCAGCCTCA	ACCTCGTCCCATGGGGTGTG
NANOG	CAACCAGACCCAGAACATCC	TTCCAAAGCAGCCTCCAAG
SLC7A11	ATTCGGACCCATTTAGTACAGG	CTCCGACATTATTCTAAACCAC

Supplementary Fig. 1 BCSCs inhibit the stemness of tumor cells. a-b, The representative images of Aldefluor data of Fig. 1a (**a**) and Fig. 1b (**b**). **c**, FACS analysis of the proportion of $CD44^{+}CD24^{-/low}$ BCSCs in mammospheres or parental T47D cells. **d**, Stem cell markers in mammospheres or parental T47D cells were determined by qRT-PCR. **e-f**, The representative images of Aldefluor data of Fig. 1e (**e**) and Fig. 1g (**f**). **g**, FACS analysis of the proportion of $CD44^{+}CD24^{-/low}$ BCSCs in MCF-7 or T47D cells cultured with control CM or BCSC CM. **h-i**, FACS analysis of the proportion of ALDH+ BCSCs in BT474 or SUM159 cells co-cultured with the respective mammosphere-enriched BCSCs or parental cells (**h**) or cultured with the respective CM derived from mammosphere-enriched BCSCs or parental cells (**i**). **j**, MCF-7 or T47D cells were cultured with CM derived from mammosphere-enriched BCSCs or parental cells for 5 days, cell viability was determined by MTT assay. Results are shown as mean ± S.D. **P*<0.05; ***P*<0.01; ****P*<0.001; ns, not significant (Unpaired two-tailed Student's t-test). Source data are provided as a Source Data file.

Supplementary Fig. 2 BCSC-secreted DKK1 inhibits CSC property. a-b, Immunoblot assessment of NICD and GLI2 (**a**) or LGR5, AXIN2 and c-MYC (**b**) protein levels in MCF-7 or T47D cells cultured with the CM obtained from respective mammosphere-enriched BCSCs or parental cells. **c**, ELISA quantification of secreted DKK1 levels in CM from sorted ALDH+ or ALDH- cells. **d**, Immunoblot assessment of β-CATENIN protein levels in MCF-7 cells treated with recombinant DKK1 (100 ng/ml). **e-f**, FACS analysis of the proportion of ALDH+ BCSCs (**e**) or mammosphere formation assay (**f**) of MCF-7 or T47D cells treated with recombinant DKK1 (100 ng/ml) or vehicle. Scale bars: 500 μm. **g**, Immunoblot assessment of DKK1 protein levels in MCF-7 or T47D cells transfected with the specific shRNAs of DKK1. **h**, The representative images of Aldefluor data of Fig. 1f. **i-j**, MCF-7 and T47D cells were cultured with the control CM or mammosphere-enriched BCSC-derived CM with or without 1 μM DKK1 Inhibitor (WAY 262611) for 48 hours, the stemness properties were subsequently analyzed by ALDEFLUOR assay (**i**) or mammosphere formation assay (**j**). Scale bars: 500 μm. Results are shown as mean ± S.D. *P<0.05; **P<0.01; ***P<0.001; ns, not significant (One-way ANOVA followed by Tukey's multiple comparison test in Supplementary Fig. 2i-2j, others unpaired two-tailed Student's t-test). Source data are provided as a Source Data file.

Supplementary Fig. 3 BCSC secreted DKK1 enhances metastatic colonization. a, Immunoblot assessment of DKK1 protein levels in SUM159 cells stably transfected with DKK1 shRNA. **b**, 1×10^6 luciferase-labeled 4TO7 cells were intravenously co-injected with 4×10^5 unlabeled mammosphere-enriched BCSCs or parental cells into the host mice. BLI was performed on the metastatic burden of 4TO7-luc cells. **c**, BLI detection of the metastasis derived from SUM159-luc cells. **d-e**, Phase-contrast images (**d**) or transwell migration and invasion assays (**e**) of MDA-MB-231 and SUM159 cell lines. Scale bar: 300 μm. **f-g**, Phase-contrast images (**f**) or transwell migration and invasion assays (**g**) of 4T1 and 4TO7 cell lines. Scale bar: 300 μm. **h**, Immunoblot assessment of DKK1 protein levels in MDA-MB-231 cells stably transfected with DKK1 shRNA. **i-j**, FACS analysis of the proportion of ALDH+ BCSCs (**i**) or transwell migration and invasion assays (**j**) of MDA-MB-231 cells transfected with DKK1 shRNA. Scale bar: 300 μm. **k**, H&E staining analysis showing muscle or fat infiltration of tumors derived from MDA-MB-231-shCONT or -shDKK1 cells. Scale bar: 300 μm. **l-m**, Tumor growth curve (**l**) or Ki-67 staining (**m**) of mice orthotopically implanted with MDA-MB-231-shCONT or -shDKK1 cells. Scale bar: 50 μm. **n**, The average number of micrometastases in lung sections from mice orthotopically implanted with MDA-MB-231-shCONT or -shDKK1 cells was plotted. **o**, BLI of the residing cells in the lung from mice intravenously injected with 1×10^6 MDA-MB-231-shCONT or -shDKK1 cells 4 hours later. **p**, H&E staining of the lung sections from mice intravenously injected with MDA-MB-231-shCONT or -shDKK1 cells. Metastatic sites are circled in red. Scale bar: 500 μm. **q-r**, IHC staining of KLF4, LIN28, NANOG

and OCT3/4 (**q**) or CCND1 and CDK4 (**r**) in lung sections from mice intravenously injected with MDA-MB-231-shCONT or -shDKK1 cells. Scale bar: 50 μm. **s**, TUNEL staining of lung sections from mice intravenously injected with MDA-MB-231-shCONT or -shDKK1 cells. Scale bar: 50 μm. **t**, Immunoblot assessment of DKK1 protein levels in SUM159 cells stably transfected with vector or DKK1 expressing plasmid. Results are shown as mean ± S.D. *P<0.05; **P<0.01; ***P<0.001; ns, not significant (Two-way ANOVA test in Supplementary Fig. 3k, others unpaired two-tailed Student's t-test). Source data are provided as a Source Data file.

Supplementary Fig. 4 DKK1 promotes mouse mammary tumor metastasis. a, Immunoblot assessment of DKK1 in 4T1 cells stably transfected with vector or DKK1 shRNA. **b**, BLI of lungs from mice orthotopically implanted with 1×10^5 4T1-shCONT or 4T1-shDKK1 cells after 4 weeks. **c**, Muscle and fat infiltration of tumors derived from 4T1-shCONT or 4T1-shDKK1 cells were analyzed by H&E staining. Scale bar: 300 μm. **d-e**, BLI of the metastatic burden of mice intravenously injected with 5×10^5 4T1-shCONT or 4T1-shDKK1 cells after 4 hours (**d**) or 7 days (**e**). **f**, Immunoblot assessment of DKK1 protein levels in 4TO7 cells stably transfected with vector or DKK1 expressing plasmid. **g**, BLI of the metastatic burden of mice intravenously injected with 1×10^6 4TO7-vector or 4TO7-DKK1 cells 3 weeks later. **h**, Kaplan-Meier plots of overall survival (OS) in gastric, lung, pancreatic or head-neck SCC stratified according to their DKK1 levels. Results are shown as mean ± S.D. $^*P<0.05$; $^{**}P<0.01$; $^{***}P<0.001$; ns, not significant (Unpaired two-tailed Student's t-test). Source data are provided as a Source Data file.

Supplementary Fig. 5 Targeting DKK1 ameliorates metastatic progression. a-b, Representative images of the 3D culturing (**a**) or immunoblot assessment of β-CATENIN levels (**b**) in MDA-MB-231 cells treating with vehicle, WAY262611 (1 μM) or Gallocyanine (5 μM). Scale bar: 300 μm. **c-d**, Body weight (**c**) and H&E staining of the kidney or liver (**d**) of BALB/c mice treated with vehicle, WAY262611 or Gallocyanine for 3 weeks. Scale Bar: 100 μm. **e**, H&E staining of the lung sections from mice intravenously injected with MDA-MB-231-luc cells. The mice were treated with vehicle, WAY262611 or Gallocyanine. Metastatic sites are indicated in red. Scale bar: 500 μm. **f-g**, IHC staining of KLF4, LIN28, NANOG and OCT3/4 (**f**) or CCND1 and CDK4 (**g**) in lung sections from mice intravenously injected with MDA-MB-231-luc cells. The mice were treated with vehicle, WAY262611 or Gallocyanine. Scale bar: 50 μm. **h**, BLI of the metastatic burden of mice intravenously injected with 5×10^5 4T1 cells. The mice were treated with vehicle or Gallocyanine for 7 days. **i**, Survival curve of BALB/c mice intravenously injected with 1×10^5 4T1 cells and treated with vehicle or Gallocyanine. **j**, BLI of the metastatic burden of NCG mice intravenously injected with 1×10^5 4T1-luc cells. The mice were treated with vehicle or Gallocyanine. Results are shown as mean ± S.D. **P*<0.05; ***P*<0.01; ****P*<0.001; ns, not significant (Unpaired two-tailed Student's t-test in Supplementary Fig. 5h and 5j, others one-way ANOVA followed by Tukey's multiple comparison test). Source data are provided as a Source Data file.

Supplementary Fig. 6 DKK1 promotes ferroptosis resistance. a, FACS analysis of ALDH+ BCSCs in MCF-7, MDA-MB-231 and 4T1 cells treated with Erastin or vehicle. **b,** Cell viability of MCF-7, MDA-MB-231 or 4T1 cells cultured with respective control CM or BCSC CM ± 5 μM Gallocyanine and treated with a graded concentration of RSL3. **c-d,** Cell viability of MCF-7, MDA-MB-231 or 4T1 cells cultured with the CM derived from parental cells or mammosphere-enriched shCONT-BCSCs or shDKK1-BCSCs and treated with a graded concentration of Erastin (**c**) or RSL3 (**d**). **e,** Cell viability of MCF-7, MDA-MB-231 or 4T1 cells were cultured with 100 ng/ml recombinant DKK1 and treated with a graded concentration of RSL3. **f,** Lipid ROS levels in MCF-7, MDA-MB-231 or 4T1 cells primed with or without 100 ng/ml DKK1, and then further with or without Erastin. **g,** Relative GSH levels in MCF-7, MDA-MB-231 or 4T1 cells primed with or without 100 ng/ml DKK1, and further treated with or without Erastin. **h,** Immunoblot assessment of DKK1 levels in MCF-7 cells treated with Erastin. **i,** IHC staining of PTGS2 in lung metastases derived from intravenously injected with MDA-MB-231 cells and treated with vehicle, WAY262611 or Gallocyanine. Scale bar: 50 μm. **j-k,** IHC staining of PTGS2 (**j**) and Ki-67 (**k**) in lung metastases derived from mice intravenously injected with 1×10^6 MDA-MB-231-shCONT or -shDKK1 cells and treated with vehicle or Liproxstatin-1 as indicated. Scale bar: 50 μm. Results are shown as mean ± S.D. **P*<0.05; ***P*<0.01; ****P*<0.001; ns, not significant (Unpaired two-tailed Student's t-test in Supplementary Fig. 6a, one-way ANOVA followed by Tukey's multiple comparison test in Supplementary Fig. 6g and 6k, others two-way ANOVA test). Source data are provided as a Source Data file.

Supplementary Fig. 7 DKK1 promotes SLC7A11 expression. a, qRT-PCR analysis of SLC7A11 mRNA levels in MCF-7 cells cultured with control CM or BCSC CM. **b,** Immunoblot assessment of SLC7A11 levels in MCF-7 or MDA-MB-231 cells transfected with SLC7A11 shRNA. **c-d,** Cell viability of MCF-7 or MDA-MB-231 cells transfected with SLC7A11 shRNA or vector and further treated with a graded concentration of Erastin (**c**) or RSL3 (**d**) for 48 hours. **e,** Kaplan-Meier plots of overall survival (OS) in the indicated population of breast cancer patients stratified according to tumor SLC7A11 expression. **f**, Immunoblot assessment of β-CATENIN, phosphorylated and total STAT3 protein levels in MDA-MB-231 cells treated with vehicle or 100 ng/ml DKK1. **g,** MCF-7-shCONT or -shCTNNB1 cells were cultured with 100ng/ml DKK1 or vehicle and treated with a graded concentration of Erastin for 48 hours. Cell viability was determined by MTT assay. **h,** Cell viability of the parental or lung metastatic derived MDA-MB-231 or 4T1 cells treated with a graded concentration of RSL3 for 48 hours. **i-j,** IHC staining for PTGS2 (**i**) and Ki-67 (**j**) in lung sections derived from 4T1 cells. The mice were treated with Erastin, Gallocyanine or combined Erastin and Gallocyanine as indicated. Scale bar: 50 μm. Results are shown as mean ± S.D. **P*<0.05; ***P*<0.01; ****P*<0.001; ns, not significant (Unpaired two-tailed Student's t-test in Supplementary Fig. 7a, one-way ANOVA followed by Tukey's multiple comparison test in Supplementary Fig. 7j, others Two-way ANOVA test). Source data are provided as a Source Data file.

Supplementary Fig. 8 β-CATENIN depletion attenuates the regulatory effect of BCSCs on CSC property. **a**, FACS analysis of the proportion of ALDH+ BCSCs in MCF-7 cells cultured with control CM, BCSC CM or shCTNNB1-BCSC CM. **b**, Schematic of BCSC-secreted DKK1 conditioned breast cancer metastatic colonization by protecting cells from ferroptosis. Results are shown as mean ± S.D. *$P<0.05$; **$P<0.01$; ***$P<0.001$; ns, not significant (One-way ANOVA followed by Tukey's multiple comparison test in Supplementary Fig. 8a). Source data are provided as a Source Data file.
