# Supplementary material for: Cancer stem cell regulated phenotypic plasticity protects metastasized cancer cells from ferroptosis
Source: Nat Commun. 2022 Mar 16;13:1371. doi: 10.1038/s41467-022-29018-9 (PMC8927306; doi:10.1038/s41467-022-29018-9)
Supplement: Supplementary file 3 — Reporting Summary [file 41467_2022_29018_MOESM3_ESM.pdf]

nature research

Corresponding author(s): Tao Zhu, Peter E. Lobie

Last updated by author(s): Feb 7, 2022

Reporting Summary

Nature Research wishes to improve the reproducibility of the work that we publish. This form provides structure for consistency and transparency in reporting. For further information on Nature Research policies, see our Editorial Policies and the Editorial Policy Checklist.

Statistics

For all statistical analyses, confirm that the following items are present in the figure legend, table legend, main text, or Methods section.

n/a	Confirmed	
[ ]	[x]	The exact sample size (*n*) for each experimental group/condition, given as a discrete number and unit of measurement
[ ]	[x]	A statement on whether measurements were taken from distinct samples or whether the same sample was measured repeatedly
[ ]	[x]	The statistical test(s) used AND whether they are one- or two-sided<br>*Only common tests should be described solely by name; describe more complex techniques in the Methods section.*
[x]	[ ]	A description of all covariates tested
[ ]	[x]	A description of any assumptions or corrections, such as tests of normality and adjustment for multiple comparisons
[ ]	[x]	A full description of the statistical parameters including central tendency (e.g. means) or other basic estimates (e.g. regression coefficient) AND variation (e.g. standard deviation) or associated estimates of uncertainty (e.g. confidence intervals)
[ ]	[x]	For null hypothesis testing, the test statistic (e.g. *F*, *t*, *r*) with confidence intervals, effect sizes, degrees of freedom and *P* value noted<br>*Give P values as exact values whenever suitable.*
[x]	[ ]	For Bayesian analysis, information on the choice of priors and Markov chain Monte Carlo settings
[x]	[ ]	For hierarchical and complex designs, identification of the appropriate level for tests and full reporting of outcomes
[x]	[ ]	Estimates of effect sizes (e.g. Cohen's *d*, Pearson's *r*), indicating how they were calculated

Our web collection on statistics for biologists contains articles on many of the points above.

Software and code

Policy information about availability of computer code

Data collection	The bioluminescent imaging was collected using a PerkinElmer IVIS Spectrum system.The IHC staining of clinical specimens were scanned by Tissue FAXS Plus microscope. Western blot and antibody array were imaged on ImageQuant LAS4000mini. Real-time PCR was run on Stratagene MX3000P. Flow cytometer BD FACS Calibur was used for FACS analysis.
Data analysis	GraphPad Prism version 8 was used for data analysis. FACS data were analyzed with FlowJo version 7. The Kaplan Meier plotter database was used to analyze the effect of DKK1 or IL8 on the survival of cancer patients.The bioluminescent imaging was quantified using Living Image 4.5 software. The IHC staining image was analysis by HistoQuest 4.0 software.The CSC frequency was calculated using online ELDA software (https://bioinf.wehi.edu.au/software/elda/).

For manuscripts utilizing custom algorithms or software that are central to the research but not yet described in published literature, software must be made available to editors and reviewers. We strongly encourage code deposition in a community repository (e.g. GitHub). See the Nature Research guidelines for submitting code & software for further information.

Data

Policy information about availability of data

All manuscripts must include a data availability statement. This statement should provide the following information, where applicable:

- Accession codes, unique identifiers, or web links for publicly available datasets
- A list of figures that have associated raw data
- A description of any restrictions on data availability

The RNA sequencing data has been deposited in GEO with the accession number: GSE156454. Source data are provided with this paper. The ChIP sequencing data used in this study was downloaded from GEO dataset: GSE31477 (https://www.ncbi.nlm.nih.gov/geo/query/acc.cgi?acc=GSE31477) and GSE64758 (https://www.ncbi.nlm.nih.gov/geo/query/acc.cgi?acc=GSE64758). The RNA sequencing data used in this study was downloaded from GEO dataset: GSE59653 (https://

www.ncbi.nlm.nih.gov/geo/query/acc.cgi?acc=GSE59653) and GSE80213 (https://www.ncbi.nlm.nih.gov/geo/query/acc.cgi?acc=GSE80213) or TCGA dataset (http://www.cbioportal.org/). All other data supporting the findings of this study are available within the article and its supplementary files.

Field-specific reporting

Please select the one below that is the best fit for your research. If you are not sure, read the appropriate sections before making your selection.

- [x] Life sciences
- [ ] Behavioural & social sciences
- [ ] Ecological, evolutionary & environmental sciences

For a reference copy of the document with all sections, see nature.com/documents/nr-reporting-summary-flat.pdf

Life sciences study design

All studies must disclose on these points even when the disclosure is negative.

Sample size	No statistical method was used to calculate sample size. Sample size was determined to be adequate based on the magnitude and consistency of measurable differences between groups. The size of animal studies is between 4 to 30, which are commonly used in similar studies in literatures (https://doi.org/10.1038/s41586-019-1170-y). The size of other studies is at least n=3 base on the stands of literatures, which enables the measurable differences between groups.
Data exclusions	No data was excluded for all experiments
Replication	As reported in the figure legends, experiments were performed at least three times with similar results, the findings were reliably reproduced.
Randomization	For all the experiments, samples/participants were allocated into experimental groups were randomly assigned into control group and test group.
Blinding	No blinding test was used in this study since the same investigator designed and performed experiments.

Behavioural & social sciences study design

All studies must disclose on these points even when the disclosure is negative.

Study description	*Briefly describe the study type including whether data are quantitative, qualitative, or mixed-methods (e.g. qualitative cross-sectional, quantitative experimental, mixed-methods case study).*
Research sample	*State the research sample (e.g. Harvard university undergraduates, villagers in rural India) and provide relevant demographic information (e.g. age, sex) and indicate whether the sample is representative. Provide a rationale for the study sample chosen. For studies involving existing datasets, please describe the dataset and source.*
Sampling strategy	*Describe the sampling procedure (e.g. random, snowball, stratified, convenience). Describe the statistical methods that were used to predetermine sample size OR if no sample-size calculation was performed, describe how sample sizes were chosen and provide a rationale for why these sample sizes are sufficient. For qualitative data, please indicate whether data saturation was considered, and what criteria were used to decide that no further sampling was needed.*
Data collection	*Provide details about the data collection procedure, including the instruments or devices used to record the data (e.g. pen and paper, computer, eye tracker, video or audio equipment) whether anyone was present besides the participant(s) and the researcher, and whether the researcher was blind to experimental condition and/or the study hypothesis during data collection.*
Timing	*Indicate the start and stop dates of data collection. If there is a gap between collection periods, state the dates for each sample cohort.*
Data exclusions	*If no data were excluded from the analyses, state so OR if data were excluded, provide the exact number of exclusions and the rationale behind them, indicating whether exclusion criteria were pre-established.*
Non-participation	*State how many participants dropped out/declined participation and the reason(s) given OR provide response rate OR state that no participants dropped out/declined participation.*
Randomization	*If participants were not allocated into experimental groups, state so OR describe how participants were allocated to groups, and if allocation was not random, describe how covariates were controlled.*

Ecological, evolutionary & environmental sciences study design

All studies must disclose on these points even when the disclosure is negative.

Study description	*Briefly describe the study. For quantitative data include treatment factors and interactions, design structure (e.g. factorial, nested, hierarchical), nature and number of experimental units and replicates.*

Research sample	*Describe the research sample (e.g. a group of tagged Passer domesticus, all Stenocereus thurberi within Organ Pipe Cactus National Monument), and provide a rationale for the sample choice. When relevant, describe the organism taxa, source, sex, age range and any manipulations. State what population the sample is meant to represent when applicable. For studies involving existing datasets, describe the data and its source.*
Sampling strategy	*Note the sampling procedure. Describe the statistical methods that were used to predetermine sample size OR if no sample-size calculation was performed, describe how sample sizes were chosen and provide a rationale for why these sample sizes are sufficient.*
Data collection	*Describe the data collection procedure, including who recorded the data and how.*
Timing and spatial scale	*Indicate the start and stop dates of data collection, noting the frequency and periodicity of sampling and providing a rationale for these choices. If there is a gap between collection periods, state the dates for each sample cohort. Specify the spatial scale from which the data are taken*
Data exclusions	*If no data were excluded from the analyses, state so OR if data were excluded, describe the exclusions and the rationale behind them, indicating whether exclusion criteria were pre-established.*
Reproducibility	*Describe the measures taken to verify the reproducibility of experimental findings. For each experiment, note whether any attempts to repeat the experiment failed OR state that all attempts to repeat the experiment were successful.*
Randomization	*Describe how samples/organisms/participants were allocated into groups. If allocation was not random, describe how covariates were controlled. If this is not relevant to your study, explain why.*
Blinding	*Describe the extent of blinding used during data acquisition and analysis. If blinding was not possible, describe why OR explain why blinding was not relevant to your study.*

Did the study involve field work? ☐ Yes ☐ No

Field work, collection and transport

Field conditions	*Describe the study conditions for field work, providing relevant parameters (e.g. temperature, rainfall).*
Location	*State the location of the sampling or experiment, providing relevant parameters (e.g. latitude and longitude, elevation, water depth).*
Access & import/export	*Describe the efforts you have made to access habitats and to collect and import/export your samples in a responsible manner and in compliance with local, national and international laws, noting any permits that were obtained (give the name of the issuing authority, the date of issue, and any identifying information).*
Disturbance	*Describe any disturbance caused by the study and how it was minimized.*

Reporting for specific materials, systems and methods

We require information from authors about some types of materials, experimental systems and methods used in many studies. Here, indicate whether each material, system or method listed is relevant to your study. If you are not sure if a list item applies to your research, read the appropriate section before selecting a response.

Materials & experimental systems

n/a	Involved in the study	
☐	☒	Antibodies
☐	☒	Eukaryotic cell lines
☒	☐	Palaeontology and archaeology
☐	☒	Animals and other organisms
☐	☒	Human research participants
☒	☐	Clinical data
☒	☐	Dual use research of concern

Methods

n/a	Involved in the study	
☐	☒	ChIP-seq
☐	☒	Flow cytometry
☒	☐	MRI-based neuroimaging

Antibodies

Antibodies used	Antibodies used for immunoblot: Mouse monoclonal anti-GLI2 (Santa Cruz Biotechnology, C-10, Cat#sc-271786, Lot#H0317, 1:1000), Mouse monoclonal anti-β-CATENIN (Santa Cruz Biotechnology, E-5, Cat#sc-7963, Lot#I2717, 1:1000), Mouse monoclonal anti-cMYC (Santa Cruz Biotechnology, 9E10, Cat#sc-40, Lot#K2116, 1:1000), Rabbit polyclonal anti-DKK1 (Proteintech, Cat#21112-1-AP, Lot#0064776, 1:1000), Rabbit monoclonal anti-NICD (Cell Signaling Technology, D1E11, Cat#3608, 1:1000), Rabbit anti-p-STAT3 (Cell Signaling Technology, Cat#9131, 1:1000), Mouse monoclonal anti-t-STAT3 (Santa Cruz Biotechnology, C-20, Cat#sc-482, Lot#C1212, 1:1000), Mouse monoclonal anti-LGR5 (Santa Cruz Biotechnology, 634J2E, Cat#sc-517661, 1:1000), Mouse monoclonal anti-AXIN2 (Santa Cruz Biotechnology, C-6, Cat#sc-25302, Lot#L0617, 1:1000), Mouse monoclonal anti-β-ACTIN (Proteintech, 2D4H5, Cat#66009-1-Ig, Lot#10004156, 1:5000), Recombinant monoclonal anti-SLC7A11 (Abcam, EPR8290(2), Cat#ab175186, 1:1000). Antibodies used for IHC and immunofluorescence: Mouse monoclonal anti-CDKN1B (Santa Cruz Biotechnology, F-8, Cat#sc-1641,

Lot#E3118, 1:200), Mouse monoclonal anti-CHEK1 (Santa Cruz Biotechnology, G-4, Cat#sc-8408, Lot#L2017, 1:200), Mouse monoclonal anti-TGFB2 (Santa Cruz Biotechnology, H-6, Cat#sc-374659, Lot#E0918, 1:200), Mouse monoclonal anti-KLF4 (Santa Cruz Biotechnology, F-8, Cat#sc-166238, 1:200), Mouse monoclonal anti-LIN28 (Santa Cruz Biotechnology, 6D1F9, Cat#sc-293120, Lot#F9472, 1:200), Mouse monoclonal anti-NANOG (Santa Cruz Biotechnology, 1E6C4, Cat#sc-293121, Lot#H2217, 1:200), Mouse monoclonal anti-OCT3/4 (Santa Cruz Biotechnology, C-10, Cat#sc-5279, Lot#H2317, 1:200), Mouse monoclonal anti-CCND1 (Santa Cruz Biotechnology, 72-13G, Cat#sc-450, Lot#K0513, 1:200), Mouse monoclonal anti-CDK4 (Santa Cruz Biotechnology, DCS-35, Cat#sc-23896, 1:200), Mouse monoclonal anti-PTGS2 (Santa Cruz Biotechnology, H-3, Cat#sc-376861, 1:200), Rabbit monoclonal anti-ALDH1A1 (Cell Signaling Technology, D9Q8E, Cat#54135, 1:200).

Antibodies used for FACS: FITC mouse anti-human CD44 (BD Pharmingen, Cat#555478, Lot#2167819), PE mouse anti-human CD24 (BD Pharmingen, Cat#555428, Lot#88658), FITC Mouse IgG2a, κ Isotype Control (BD Pharmingen, Cat#555573), PE Mouse IgG2a, κ Isotype Control (BD Pharmingen, Cat#555574).

Antibodies used for ChIP: Mouse monoclonal anti-TCF4 (Santa Cruz Biotechnology, C-8, Cat#sc-393407), Mouse monoclonal anti-β-CATENIN (Santa Cruz Biotechnology, E-5, Cat#sc-7963).

Antibodies used for in vivo study: anti-asialo-GM1 antibody (Wako, Cat#986-10001, Lot#EBF6552).

Validation

All antibodies are commercially available and are stated to be tested by the manufacturer for species reactivity to human and mouse. Information in CST, AbCam, Santa Cruz and Proteintech certificate of analyses. CST, Proteintech, Abcam and Santa Cruz antibodies are knock-out/knockdown validated or validated in cells with established levels of protein expression. The statements and validation data for each primary antibody for the species and application are also available on the manufacturers website. In addition antibodies were monitored in house to detect protein of the reported size by western blot in over expression and/or knock down experiments.

Eukaryotic cell lines

Policy information about cell lines

Cell line source(s)

MCF-7, T47D, BT474, MDA-MB-231 and 4T1 cells were obtained from ATCC. SUM159 was a kind gift from Suling Liu lab (Fudan University, China). HEK293T was from Dr. Ping Gao (USTC, China). 4TO7 was from Dr. Lianfeng Zhang (PUMC, China). The original commercial source of HEK293T and 4TO7 cells were from ATCC, SUM159 cells were from Asterland Bioscience.

Authentication

STR fingerprint analysis

Mycoplasma contamination

All cell lines in our laboratory are routinely tested for mycoplasma contamination and cells used in this study are negative for mycoplasma

Commonly misidentified lines
(See ICLAC register)

No cell line used in the paper is listed in ICLAC database.

Palaeontology and Archaeology

Specimen provenance

Provide provenance information for specimens and describe permits that were obtained for the work (including the name of the issuing authority, the date of issue, and any identifying information).

Specimen deposition

Indicate where the specimens have been deposited to permit free access by other researchers.

Dating methods

If new dates are provided, describe how they were obtained (e.g. collection, storage, sample pretreatment and measurement), where they were obtained (i.e. lab name), the calibration program and the protocol for quality assurance OR state that no new dates are provided.

☐ Tick this box to confirm that the raw and calibrated dates are available in the paper or in Supplementary Information.

Ethics oversight

Identify the organization(s) that approved or provided guidance on the study protocol, OR state that no ethical approval or guidance was required and explain why not.

Note that full information on the approval of the study protocol must also be provided in the manuscript.

Animals and other organisms

Policy information about studies involving animals; ARRIVE guidelines recommended for reporting animal research

Laboratory animals

The 5-week old female BALB/c nude, BALB/c and NOD-SCID mice were purchased from SLAC laboratory animal (Shanghai, China). The 5-week old female NCG mice were purchased from Gempharmatech (Nanjing, China). The MMTV-PyMT mice were from Dr. Zhenye Yang (USTC). All mice were housed under the SPF environment with a 12h light-dark cycle at 22–24°C with 50%-60% humidity.

Wild animals

The study did not involve wild animals.

Field-collected samples

The study did not involve samples collected from field.

Ethics oversight

All animal experiments were approved by the Institutional Animal Care and Use Committee, University of Science and Technology of China (USTCACUC1801035).

Note that full information on the approval of the study protocol must also be provided in the manuscript.

Human research participants

Policy information about studies involving human research participants

Population characteristics	The specimens used in this study were collected from the First Affiliated Hospital of USTC (Hefei, Anhui, China). The specimens used in this study were collected from 15 female breast cancer patients (stage from II to III), with the age range from 43-71.
Recruitment	None selection had been made. The participants were recruited from 2018 to 2019 without any self-selection bias.
Ethics oversight	The clinical research protocol was approved by the Biomedical Ethics Committee of USTC (2020-P-054).

Note that full information on the approval of the study protocol must also be provided in the manuscript.

Clinical data

Policy information about clinical studies

All manuscripts should comply with the ICMJEguidelines for publication of clinical research and a completedCONSORT checklist must be included with all submissions.

Clinical trial registration	*Provide the trial registration number from ClinicalTrials.gov or an equivalent agency.*
Study protocol	*Note where the full trial protocol can be accessed OR if not available, explain why.*
Data collection	*Describe the settings and locales of data collection, noting the time periods of recruitment and data collection.*
Outcomes	*Describe how you pre-defined primary and secondary outcome measures and how you assessed these measures.*

Dual use research of concern

Policy information about dual use research of concern

Hazards

Could the accidental, deliberate or reckless misuse of agents or technologies generated in the work, or the application of information presented in the manuscript, pose a threat to:

No	Yes	
☐	☐	Public health
☐	☐	National security
☐	☐	Crops and/or livestock
☐	☐	Ecosystems
☐	☐	Any other significant area

Experiments of concern

Does the work involve any of these experiments of concern:

No	Yes	
☐	☐	Demonstrate how to render a vaccine ineffective
☐	☐	Confer resistance to therapeutically useful antibiotics or antiviral agents
☐	☐	Enhance the virulence of a pathogen or render a nonpathogen virulent
☐	☐	Increase transmissibility of a pathogen
☐	☐	Alter the host range of a pathogen
☐	☐	Enable evasion of diagnostic/detection modalities
☐	☐	Enable the weaponization of a biological agent or toxin
☐	☐	Any other potentially harmful combination of experiments and agents

ChIP-seq

Data deposition

☐ Confirm that both raw and final processed data have been deposited in a public database such as GEO.

☐ Confirm that you have deposited or provided access to graph files (e.g. BED files) for the called peaks.

Data access links<br>*May remain private before publication.*	*For "Initial submission" or "Revised version" documents, provide reviewer access links. For your "Final submission" document, provide a link to the deposited data.*
Files in database submission	*Provide a list of all files available in the database submission.*
Genome browser session<br>(e.g. UCSC)	*Provide a link to an anonymized genome browser session for "Initial submission" and "Revised version" documents only, to enable peer review. Write "no longer applicable" for "Final submission" documents.*

Methodology

Replicates	*Describe the experimental replicates, specifying number, type and replicate agreement.*
Sequencing depth	*Describe the sequencing depth for each experiment, providing the total number of reads, uniquely mapped reads, length of reads and whether they were paired- or single-end.*
Antibodies	*Describe the antibodies used for the ChIP-seq experiments; as applicable, provide supplier name, catalog number, clone name, and lot number.*
Peak calling parameters	*Specify the command line program and parameters used for read mapping and peak calling, including the ChIP, control and index files used.*
Data quality	*Describe the methods used to ensure data quality in full detail, including how many peaks are at FDR 5% and above 5-fold enrichment.*
Software	*Describe the software used to collect and analyze the ChIP-seq data. For custom code that has been deposited into a community repository, provide accession details.*

Flow Cytometry

Plots

Confirm that:

- [x] The axis labels state the marker and fluorochrome used (e.g. CD4-FITC).
- [x] The axis scales are clearly visible. Include numbers along axes only for bottom left plot of group (a 'group' is an analysis of identical markers).
- [x] All plots are contour plots with outliers or pseudocolor plots.
- [x] A numerical value for number of cells or percentage (with statistics) is provided.

Methodology

Sample preparation	For ALDEFLUOR assay, cells were collected and resuspended with assay buffer, and then stained with ALDEFLUOR reagent at 37°C for 30 mins, the control samples stained with ALDEFLUOR reagent and DEAB. Cells were centrifuged and resuspended with assay buffer and directly run on a flow cytometer.<br>For CD44/CD24 staining, cells were collected and resuspended with PBS, and then stained with the antibodies at 4°C for 30 mins and directly run on a flow cytometer.<br>For lipid peroxidation detection, cells were seeded in 6-well plates and cultured with the respective treatments for 48 hours. cells were incubated with BODIPY-C11 for 30 min at 37 °C. Cells were then collected by trypsinization and washed twice with PBS, then analyzed immediately with a flow cytometer.
Instrument	BD FACS Calibur
Software	All data were analyzed with FlowJo V7.
Cell population abundance	When cells were sorted or enriched, the purity was confirmed by flow cytometry and in each case was above 90% purity.
Gating strategy	Using the FSC/SSC gating, debris was removed by gating on the main cell population. For ALDEFLUOR assay, positive threshold for each cell line was defined on the basis of DEAB group.For BODIPY-C11 staining, positive threshold for each cell line was defined on the basis of control sample. Identical positivity threshold was applied to all samples within cell line.

- [x] Tick this box to confirm that a figure exemplifying the gating strategy is provided in the Supplementary Information.

Magnetic resonance imaging

Experimental design

Design type	*Indicate task or resting state; event-related or block design.*
Design specifications	*Specify the number of blocks, trials or experimental units per session and/or subject, and specify the length of each trial or block (if trials are blocked) and interval between trials.*

Behavioral performance measures	*State number and/or type of variables recorded (e.g. correct button press, response time) and what statistics were used to establish that the subjects were performing the task as expected (e.g. mean, range, and/or standard deviation across subjects).*

Acquisition

Imaging type(s)	*Specify: functional, structural, diffusion, perfusion.*
Field strength	*Specify in Tesla*
Sequence & imaging parameters	*Specify the pulse sequence type (gradient echo, spin echo, etc.), imaging type (EPI, spiral, etc.), field of view, matrix size, slice thickness, orientation and TE/TR/flip angle.*
Area of acquisition	*State whether a whole brain scan was used OR define the area of acquisition, describing how the region was determined.*

Diffusion MRI ☐ Used ☐ Not used

Preprocessing

Preprocessing software	*Provide detail on software version and revision number and on specific parameters (model/functions, brain extraction, segmentation, smoothing kernel size, etc.).*
Normalization	*If data were normalized/standardized, describe the approach(es): specify linear or non-linear and define image types used for transformation OR indicate that data were not normalized and explain rationale for lack of normalization.*
Normalization template	*Describe the template used for normalization/transformation, specifying subject space or group standardized space (e.g. original Talairach, MNI305, ICBM152) OR indicate that the data were not normalized.*
Noise and artifact removal	*Describe your procedure(s) for artifact and structured noise removal, specifying motion parameters, tissue signals and physiological signals (heart rate, respiration).*
Volume censoring	*Define your software and/or method and criteria for volume censoring, and state the extent of such censoring.*

Statistical modeling & inference

Model type and settings	*Specify type (mass univariate, multivariate, RSA, predictive, etc.) and describe essential details of the model at the first and second levels (e.g. fixed, random or mixed effects; drift or auto-correlation).*
Effect(s) tested	*Define precise effect in terms of the task or stimulus conditions instead of psychological concepts and indicate whether ANOVA or factorial designs were used.*

Specify type of analysis: ☐ Whole brain ☐ ROI-based ☐ Both

Statistic type for inference (See Eklund et al. 2016)	*Specify voxel-wise or cluster-wise and report all relevant parameters for cluster-wise methods.*
Correction	*Describe the type of correction and how it is obtained for multiple comparisons (e.g. FWE, FDR, permutation or Monte Carlo).*

Models & analysis

n/a	Involved in the study
☐	☐ Functional and/or effective connectivity
☐	☐ Graph analysis
☐	☐ Multivariate modeling or predictive analysis

Functional and/or effective connectivity	*Report the measures of dependence used and the model details (e.g. Pearson correlation, partial correlation, mutual information).*
Graph analysis	*Report the dependent variable and connectivity measure, specifying weighted graph or binarized graph, subject- or group-level, and the global and/or node summaries used (e.g. clustering coefficient, efficiency, etc.).*
Multivariate modeling and predictive analysis	*Specify independent variables, features extraction and dimension reduction, model, training and evaluation metrics.*
